# Extra-cellular expansion in the normal, non-infarcted myocardium is associated with worsening of regional myocardial function after acute myocardial infarction

**DOI:** 10.1186/s12968-017-0384-0

**Published:** 2017-09-25

**Authors:** Pankaj Garg, David A. Broadbent, Peter P. Swoboda, James R.J. Foley, Graham J. Fent, Tarique A. Musa, David P. Ripley, Bara Erhayiem, Laura E. Dobson, Adam K. McDiarmid, Philip Haaf, Ananth Kidambi, Saul Crandon, Pei G. Chew, R. J. van der Geest, John P. Greenwood, Sven Plein

**Affiliations:** 1 Division of Biomedical Imaging, Leeds Institute of Cardiovascular and Metabolic Medicine (LICAMM) & Multidisciplinary Cardiovascular Research Centre, University of Leeds, Leeds, LS2 9JT UK; 2 Medical Physics and Engineering, Leeds Teaching Hospitals NHS Trust, Leeds, UK; 3 Division of Image Processing, Leiden University Medical Centre, Leiden, The Netherlands

**Keywords:** Acute myocardial infarction, CT and MRI, Cardiovascular imaging agents/techniques, Extracellular matrix

## Abstract

**Background:**

Expansion of the myocardial extracellular volume (ECV) is a surrogate measure of focal/diffuse fibrosis and is an independent marker of prognosis in chronic heart disease. Changes in ECV may also occur after myocardial infarction, acutely because of oedema and in convalescence as part of ventricular remodelling. The objective of this study was to investigate changes in the pattern of distribution of regional (normal, infarcted and oedematous segments) and global left ventricular (LV) ECV using semi-automated methods early and late after reperfused ST-elevation myocardial infarction (STEMI).

**Methods:**

Fifty patients underwent cardiovascular magnetic resonance (CMR) imaging acutely (24 h–72 h) and at convalescence (3 months). The CMR protocol included: cines, T2-weighted (T2 W) imaging, pre−/post-contrast T1-maps and LGE-imaging. Using T2 W and LGE imaging on acute scans, 16-segments of the LV were categorised as normal, oedema and infarct. 800 segments (16 per-patient) were analysed for changes in ECV and wall thickening (WT).

**Results:**

From the acute studies, 325 (40.6%) segments were classified as normal, 246 (30.8%) segments as oedema and 229 (28.6%) segments as infarct. Segmental change in ECV between acute and follow-up studies (Δ ECV) was significantly different for normal, oedema and infarct segments (0.8 ± 6.5%, −1.78 ± 9%, −2.9 ± 10.9%, respectively; *P* < 0.001). Normal segments which demonstrated deterioration in wall thickening at follow-up showed significantly increased Δ ECV compared with normal segments with preserved wall thickening at follow up (1.82 ± 6.05% versus −0.10 ± 6.88%, *P* < 0.05).

**Conclusion:**

Following reperfused STEMI, normal myocardium demonstrates subtle expansion of the extracellular volume at 3-month follow up. Segmental ECV expansion of normal myocardium is associated with worsening of contractile function.

**Electronic supplementary material:**

The online version of this article (10.1186/s12968-017-0384-0) contains supplementary material, which is available to authorized users.

## Background

Following ST-elevation myocardial infarction (STEMI), even with immediate mechanical reperfusion therapy, 24–30% patients develop adverse left ventricular (LV) remodelling [1, 2]. LV remodelling is a predictor of heart failure, and hence is associated with morbidity and mortality. Post infarct, the acute loss of myocardial function results in an abrupt increase in LV loading conditions that induces a unique pattern of remodelling involving the normal (non-infarcted non-oedematous myocardium), oedematous (injured with oedematous myocardium) and infarcted myocardium [3].

The chronic phase of LV remodelling involves compensatory myocyte hypertrophy and alterations in ventricular geometry to distribute the increased wall stresses more evenly [4]. Early pre-clinical studies have speculated that the ‘normal’ non-infarcted myocardium also undergoes changes due to increased wall stress [5, 6]. There is limited evidence to support these concepts in humans. Additionally, it remains unknown if these changes in tissue composition of normal myocardium have any impact on regional contractility.

Cardiovascular magnetic resonance (CMR) offers comprehensive multi-parametric structural and functional assessment in patients with STEMI [7]. Using early gadolinium enhancement (EGE) and late gadolinium enhancement (LGE) imaging, accurate assessment of infarct characteristics (infarct size, transmurality of scar, presence of microvascular obstruction and LV thrombus) can be made. T2-weighted (T2 W) imaging allows to diagnose and quantify the extent of myocardial oedema following acute ischaemic injury [8, 9]. Native T1-mapping can also detect acute ischaemia and combined with post contrast T1 mapping allows quantification of the extra-cellular volume (ECV) [10–13].

This study aimed to investigate whether ECV expansion of normal myocardium occurs after STEMI and whether it is associated with a reduction in contractile function between baseline and follow up assessment. We also sought to determine the baseline CMR parameters that are most strongly associated with segmental functional change at follow up.

## Methods

### Study population

**Fig. 1:**
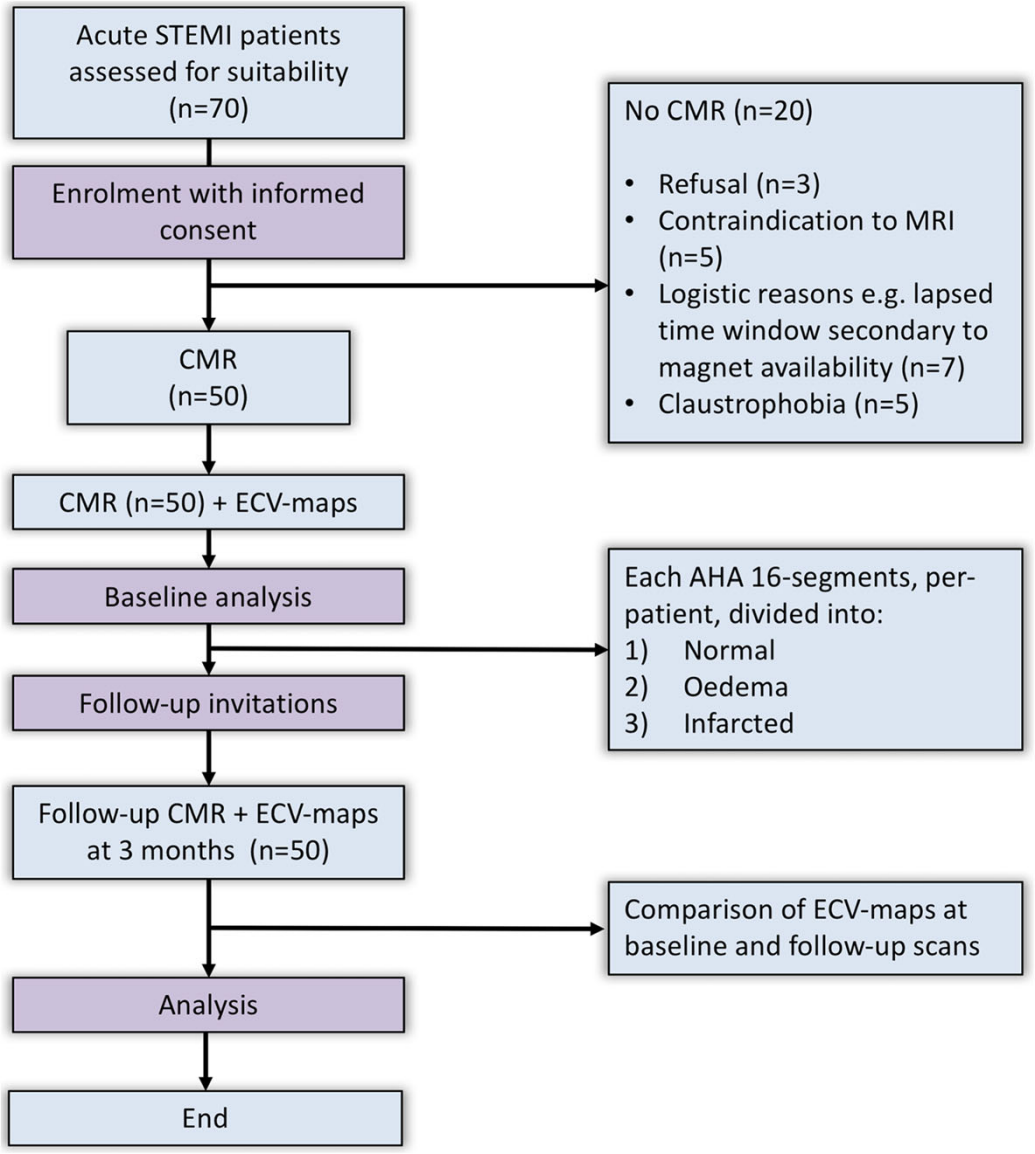
Study design

Patients with acute STEMI were prospectively enrolled from a single UK tertiary centre. The study design is detailed in Fig. 1.

The inclusion criteria were as follows: patients with first time acute STEMI treated by primary percutaneous coronary intervention (PPCI) within 12-h of onset of chest pain. Acute STEMI was defined as per current international guidelines [14]. Exclusion criteria included: previous myocardial infaction, coronary artery bypass grafting, known cardiomyopathy, estimated glomerular filtration rate < 30 ml/min/1.73 m2, haemodynamic instability (requiring on-going intravenous therapy or respiratory support) and contraindication to CMR imaging. After revascularization, all patients received standard post-myocardial infarction secondary prevention therapy at the discretion of the treating physician, and were enrolled in a cardiac rehabilitation programme if they were deemed suitable [15].

### Cardiac catheterization

Coronary angiography and revascularisation were performed in a standard fashion as per current best practice guidelines [15, 16]. TIMI flow grades were assessed visually as described previously following coronary angioplasty [17].

### Cardiovascular magnetic resonance

All patients underwent CMR imaging at either 1.5 Tesla (Ingenia CV, Philips Healthcare, Best, The Netherlands) or 3.0 Tesla (Achieva TX, Philips Healthcare, Best, The Netherlands). The choice of field strength was arbitrary and was predominantly dictated by availability of the scanners. A dedicated cardiac phased array receiver coil was used (1.5 T: 24-channel equipped with d-stream; 3 T: 32-channel). Acute CMR imaging was scheduled within 72-h of the index presentation and patients were invited to attend for a further CMR study at 3-months follow-up. The same field strength scanners were used for respective patients as for the acute scans.

### Image acquisition

Cine, T2 W-imaging, EGE and LGE imaging were performed in contiguous stacks of short-axis slices covering the entire LV for each acquisition. The same slice geometry, position and 10 mm slice thickness were used for all pulse sequences.

### T1-maps acquisition

Native and post-contrast T1-maps were planned using the ‘3-of-5’ approach [18]. Post-contrast T1-maps were timed at 15 min after contrast administration and LGE-imaging was performed at 16–20 min.

### Image analysis

For each pulse sequence, images with artefact were repeated until any artefact was removed or minimized. The highest quality images were used for analysis. Cine, T2 W-images, EGE-images and LGE-images were evaluated offline using MASS research software (Version 2016EXP, Leiden University Medical Centre, Leiden, The Netherlands).

Basic CMR image analysis, T1-maps quality assurance checks, pulse sequence parameters and imaging protocol are described in the online Additional file 1.

### Categorising of left ventricular segments

Sixteen segments of the LV excluding the apex, adapted from the 17 segments of the American Heart Association (AHA) model [19], were visually assessed on acute CMR scans and labelled as 1) normal segment (no oedema on T2 W-imaging and no infarct on LGE-imaging), 2) oedema segment (predominantly oedema on T2 W-imaging with no infarct on LGE-imaging 3) infarct segment (presence of any infarction on LGE-imaging with/without oedema on T2 W-imaging).

### Myocardial wall thickening analysis

Segmental myocardial systolic wall thickening (WT) analysis was performed for each slice based on endo- and epi-cardial contours. For each segment, end-diastolic and end-systolic wall thickness (EDWT and ESWT, respectively [mm]) were recorded as per previously published literature [20]. This method has demonstrated high intra-inter-observer agreement [21]. Myocardial systolic segmental WT ([mm]) was calculated as absolute change in EDWT and ESWT. For each LV segment, delta change of WT was derived. Functional improvement of segments was defined as positive delta change of WT from baseline to follow-up and vice versa. Additionally, radial strain was computed using endo−/epi- cardial contours through-out the complete cardiac cycle as per previously described methods [22].

### Extracellular volume map analysis

ECV maps were generated for the 3 slices (base, mid and apex) from pre−/post-contrast T1-maps and haematocrit as per the standard techniques [10]. The endocardial and epicardial contours were outlined to define myocardium in the 3 slices. Microvascular obstruction (MVO) contours were imported from EGE imaging. Mean ECV values were generated for each segment of the 16 segments excluding the MVO in the infarct zone. Left ventricular global ECV was calculated by multiplication of the averaged per-patient ECV values (for all the 16 segments) and indexed LV end-diastolic volume (LVEDV). Similarly, left ventricular myocyte cell volume was calculated by using the previously published formula: (1-*global ECV*)*(*indexed* LVEDV) [23]. For each patient, mean ECV values for different types of segments (normal, oedema and infarct) were generated on both acute and follow-up studies. For each patient, increase or decrease of ECV of all non-infarcted, healthy myocardium was defined as the change in the mean ECV of all segments and this was investigated for its association with patient characteristics (Table 1).

**Table 1:** Clinical and angiographic characteristics in patients with change of normal segment ECV per-patient between acute and follow-up scan

	Normal segment ECV ↓ (*n* = 22)		Normal segment ECV ↑ (*n* = 28)		
	Mean/Median/Count	SD/ 25%–75%/%	Mean/Median/Count	SD/25%–75%/%	*p*-value
Patient Demographics					
Age, yrs	60	11	58	11	0.39
Sex (Male)^c^	20	40	22	44	0.24
Smoker^c^	14	28	16	32	0.65
Hypertension^c^	5	10	3	6	0.25
Hyperlipidaemia^c^	9	18	8	16	0.37
Diabetes Mellitus^c^	4	8	2	4	0.24
Stroke^c^	1	2	0	0	0.26
Presenting Characteristics					
Systolic Blood Pressure, mmHg	137	24	134	36	0.88
Heart rate, beats/min	73	14	74	16	0.95
Time from onset of CP to reperfusion, min^c^	261	158–454	222	149–344	0.43
Heart Failure Killip Class^a^					
I^c^	20	40	26	52	0.80
II^c^	1	2	2	4	0.70
III-IV^c^	1	2	0	0	0.26
Ventricular fibrillation at presentation^c^	0	0	3	6	0.11
Angiographic Characteristics					
Number of diseased arteries^b^					
Single vessel disease^c^	16	30	16	32	0.26
Two vessel disease^c^	5	10	5	10	0.67
Three vessel disease^c^	1	2	7	14	**0.05**
Culprit Vessel					
Left main stem^c^	0	0	1	2	0.37
Left anterior descending^c^	14	28	15	30	0.48
Left circumflex^c^	2	4	2	4	0.80
Right coronary^c^	6	12	11	22	0.38
QRS duration, msec^c^	90	86–110	94	82–100	0.86
TIMI coronary flow pre-PCI (<2)^c^	18	36	26	52	0.15
TIMI coronary flow post-PCI (<3)^c^	2	4	2	4	0.77
Laboratory results					
White blood cells^c^, ×10^9^/l	11	10–15	11	10–13	0.86
Estimated Glomerular Filtration Rate^c^, ml/min/1.73m^2^	88	77–90	90	78–90	0.47
Creatine kinase^c^, U/l	1493	786–2400	1584	867–2570	0.75
Troponin^c^,	50,000	16,441–50,000	50,000	48,335–50,000	0.34
HBA1c^c^, mmol/mol	41	36–44	40	36–44	0.77
Infarct characteristics					
Infarct size, volume in %	30	17	26	12	0.42
Area at risk, volume in %	49	19	46	17	0.46
Presence of Microvascular Obstruction (MVO)	13	59	16	57	0.89

^a^Killip classification of heart failure after acute myocardial infarction: class I = no heart failure; class II = pulmonary rales or crepitations, a third heart sound, and elevated jugular venous pressure; class III = acute pulmonary edema; and class IV = cardiogenic shock

^b^Multi-vessel coronary artery disease was defined according to the number of stenoses of at least 50% of the reference vessel diameter by visual assessment and whether or not there was left main stem involvement

Abbreviations: BMI = body mass index; CMR = cardiac magnetic resonance; CP = chest pain; ECV = extracellular volume; HBA1c = glycated haemoglobin; PCI = percutaneous coronary intervention; STEMI = ST-segment elevation myocardial infarction; TIMI = Thrombolysis In Myocardial Infarction

^c^Non-normally distributed

Adverse LV remodelling was defined as an absolute increase of LV end-systolic volumes >15% at 3 months follow-up [24, 25].

### Intra−/inter-observer segmental ECV assessment

For intra−/inter-observer assessments, 48 segments were selected from three randomly chosen scans. To test the inter-observer reliability of segmental ECV values, two blinded observers carried out independent segmentation of ECV-maps (PG and SC) in these segments. To test intra-observer reliability, one observer (PG) undertook a second blinded analysis after three months.

### Statistical analysis

Statistical analysis was performed using SPSS® Statistics 21.0 (International Business Machines, Inc., Chicago, Illinois, USA). Normality of quantitative data was established using the Shapiro-Wilk test. Normally distributed continuous variables are expressed as mean ± SD and non-normally distributed are expressed as median (25th–75th quartile ranges). Demographic comparisons between patients with rise and fall of ECV at follow-up, in the normal myocardial segments, were performed with an independent samples t-test for normally distributed variables and by Mann-Whitney independent t-test for not normally distributed. For paired comparison in Table 2, Wilcoxon test was used. A repeated-measures analysis of variance (ANOVA) was performed on demographic and global ECV (rise/fall) at follow-up. Linear regression was used to investigate which baseline study parameter was most strongly associated with number of segments with functional recovery at follow-up. Univariate analysis was performed for each variable separately. Step-wise multivariate linear regression was used for parameters with statistical significance from one-way analysis (*p* < 0.1). Intra−/inter-observer agreement was assessed by investigating the coefficient of variability (CV), concordance correlation coefficient (CCC), precision and accuracy. All statistical tests were 2-tailed; *p* values <0.05 were considered significant.

**Table 2:** Baseline and follow-up CMR parameters

	Acute CMR		Follow-up CMR		*P*-value^a^
	Median	25%–75% quartiles	Median	25%–75% quartiles	
Baseline CMR characteristics					
LVEDVi, ml/m2	79.1	71–87	80.45	70–89	0.41
LVESVi, ml/m2	42	36–50	38.7	31–47	0.02
LV MASSi, grams/m2	55.45	48–64	48.55	43–55	<0.0001
EF, %	45.65	36–51	52.35	43–59	<0.0001
IS, volume in %	23.9	17–38	13.75	9–27	<0.0001
Segmental myocardial tissue composition and function					
	Median	25% - 75% Quartiles	Median	25% - 75% Quartiles	
Normal ECV, % (*n* = 325)	27.5	24.8–3 0.5	27.7	25.2–32	0.03
Oedema ECV, % (*n* = 246)	34	29–39.3	32	28–37	0.0002
Infarct ECV, % (*n* = 229)	44	37.4–49	41	33.6–46	<0.0001
Normal WT (mm) (*n* = 325)	3.7	2.7–4.8	3.8	2.0–4.7	0.81
Oedema WT (mm) (*n* = 246)	2.9	1.8–4.3	3.3	2.4–4.3	0.02
Infarct WT (mm) (*n* = 229)	1.6	0.47–2.8	2.4	1.1–3.6	<0.0001
Normal EDT (mm)	7.3	6–8	6.7	6–7.5	<0.0001
Oedema EDT (mm)	7.4	6–8.5	6.5	5–7.6	<0.0001
Infarct EDT (mm)	7.9	7–9	6.4	56–7.6	<0.0001
Global myocardial tissue composition					
Total LV myocyte volume, mL/m2	51.2	45.7–57.4	53.6	46.3–61	0.14
Total LV extracellular matrix, ml/m2	25.8	22.7–32.8	26.1	21.8–32.8	0.68

LV measurements are indexed to body surface area (BSA), infarct volumes are unindexed. LVEDVi = Left ventricular end-diastolic volume (indexed), LVESVi = Left ventricular end-systolic volume (indexed), LVMi = Left ventricular mass (indexed), RS = peak systolic radial strain (%)

^a^Wilcoxon test (paired samples)

### Sample size calculations

We used data of remote zone ECV from published literature using to inform the sample size calculations for this study. In a previous study, delta remote zone ECV in patients with/without adverse LV remodelling were: 0.9 ± 2.2% (with adverse LV remodelling, *n* = 8) versus 0.9 ± 0.9% (without adverse LV remodelling, *n* = 32) [26]. Using these data, as per the mean comparison method described by Machin et al., 295 normal segments were needed to investigate functional changes. [27]. Presuming 40% of myocardium is normal post STEMI, the study thus needed to recruit at least 46 patients to give a power of 80% at an alpha of 0.05.

## Results

### Patient characteristics

Seventy patients were considered for inclusion, of which 50 had baseline and follow-up CMR (Fig. 1). Acute scans were performed at a median of 48 h after the index presentation. 32 patients had CMR at 1.5 Tesla and 18 patients had CMR at 3 Tesla. All 50 patients were included in the statistical analysis. Clinical and patient demographics are detailed in Table 1.

### Per-patient analysis

Patients were categorised into two groups depending on the rise/fall of normal myocardial ECV between baseline and follow-up studies (Table 1). Twenty-eight patients (56%) demonstrated an increase in average normal myocardial ECV at follow-up when compared to acute ECV. No significant differences were seen in baseline demographics between the two groups of patients with a rise or fall of delta-ECV. Patients with triple vessel disease were more prevalent in the group that showed a rise in ECV in normal segments (1 patient versus 7 patients, *P* = 0.05). There were no differences relating to the field strength of the scanner that was use and the 3 T patients, 10 patients (55.6%) demonstrated ECV expansion in normal myocardial versus 18 (56.2%) on the 1.5 T (*P* = 0.96 for comparison of 3 T and 1.5 T). LV mass reduced significantly from baseline to follow-up, but global ECV and myocyte cell volume showed no significant change (Table 2).

### Per-segment analysis

800 segments were analysed acutely and at follow-up. From the acute studies, 325 (40.6%) segments were classified as normal, 246 (30.8%) segments were classified as oedema and 229 (28.6%) segments were classified as infarct segments. Myocardial oedema was only seen in the peri-infarct zone of the culprit vessel.

### Intra−/inter-observer checks for segmental ECV

For the 48 segments which were analysed, intra-observer CV was 6%, with excellent CCC (0.94, 95% CI 0.90–97), high precision (0.95) and accuracy (0.99). For the inter-observer analysis, CV was 7%, with good CCC (0.92, 95% CI 0.87–95) and high precision (0.93) and accuracy (0.99).

### Pattern of ECV change

Oedema and infarct segments demonstrated significant reductions in ECV at follow-up (Table 2). Conversely, there was a smaller but statistically significant rise in the ECV of normal myocardium at follow-up (*P* = 0.03).

**Fig. 2:**
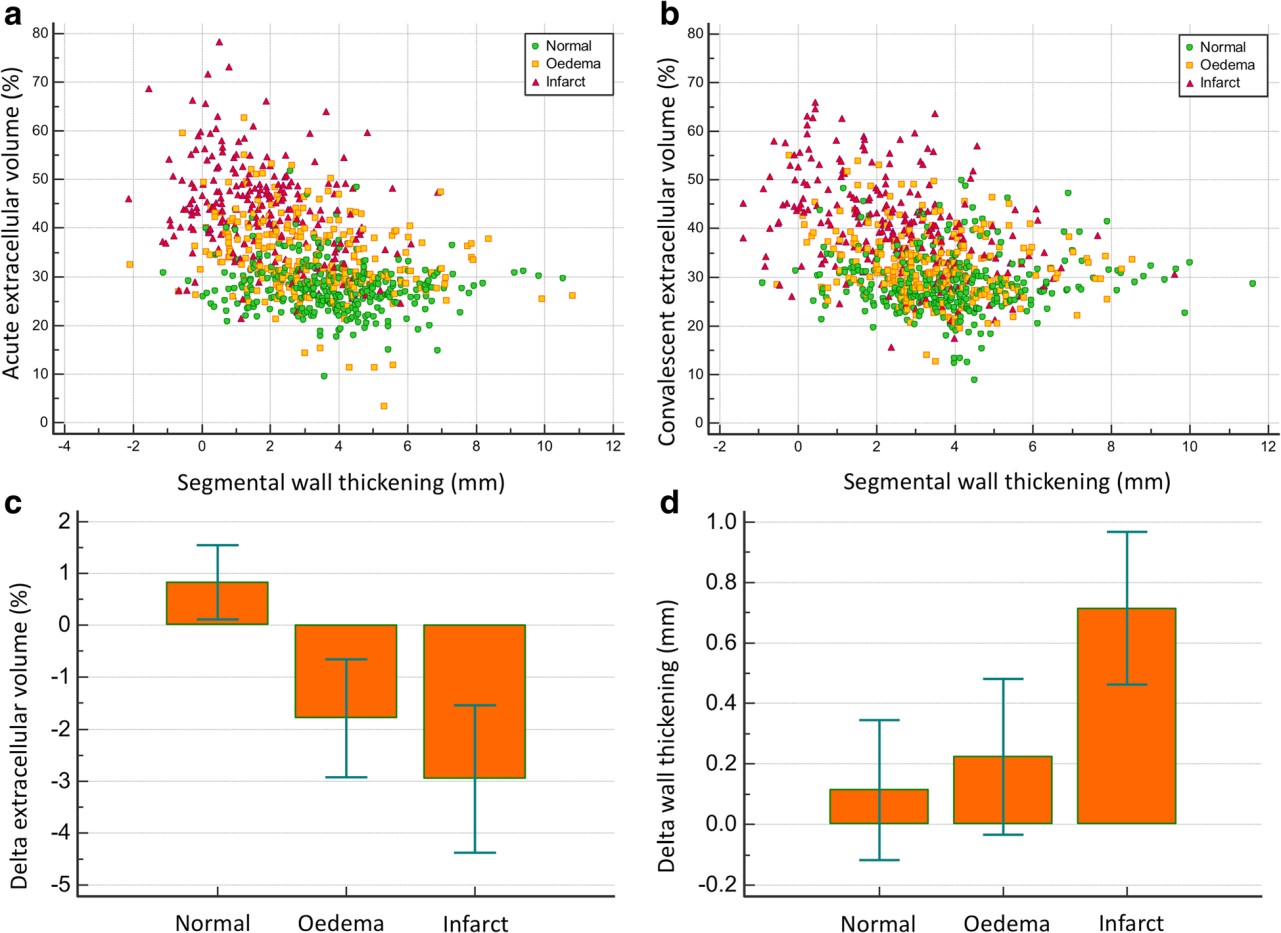
Panel A and B: Scatter plot of 800 segmental ECV values and wall thickening acutely (**a**) and at follow-up (**b**). Panel C and D: Cluster bar chart of delta ECV (**c**) and wall thickening (**d**)

Segmental change in ECV between acute and follow-up studies was significantly different for normal, oedema and infarct segments (0.8 ± 6.5% versus −1.78 ± 9% or −2.9 ± 10.9%; *P* < 0.001) (Fig. 2).

**Fig. 3:**
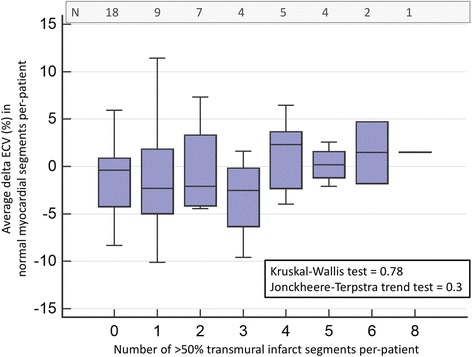
Cluster bar chart of delta ECV change in 800 segments divided into two categories: segments with functional deterioration and functional improvement. Error bars represent standard deviation

Temporal changes in normal segmental ECV did not demonstrate any significant association with number of >50% transmural scar segments (Fig. 3).

### Segmental ECV and WT

**Fig. 4:**
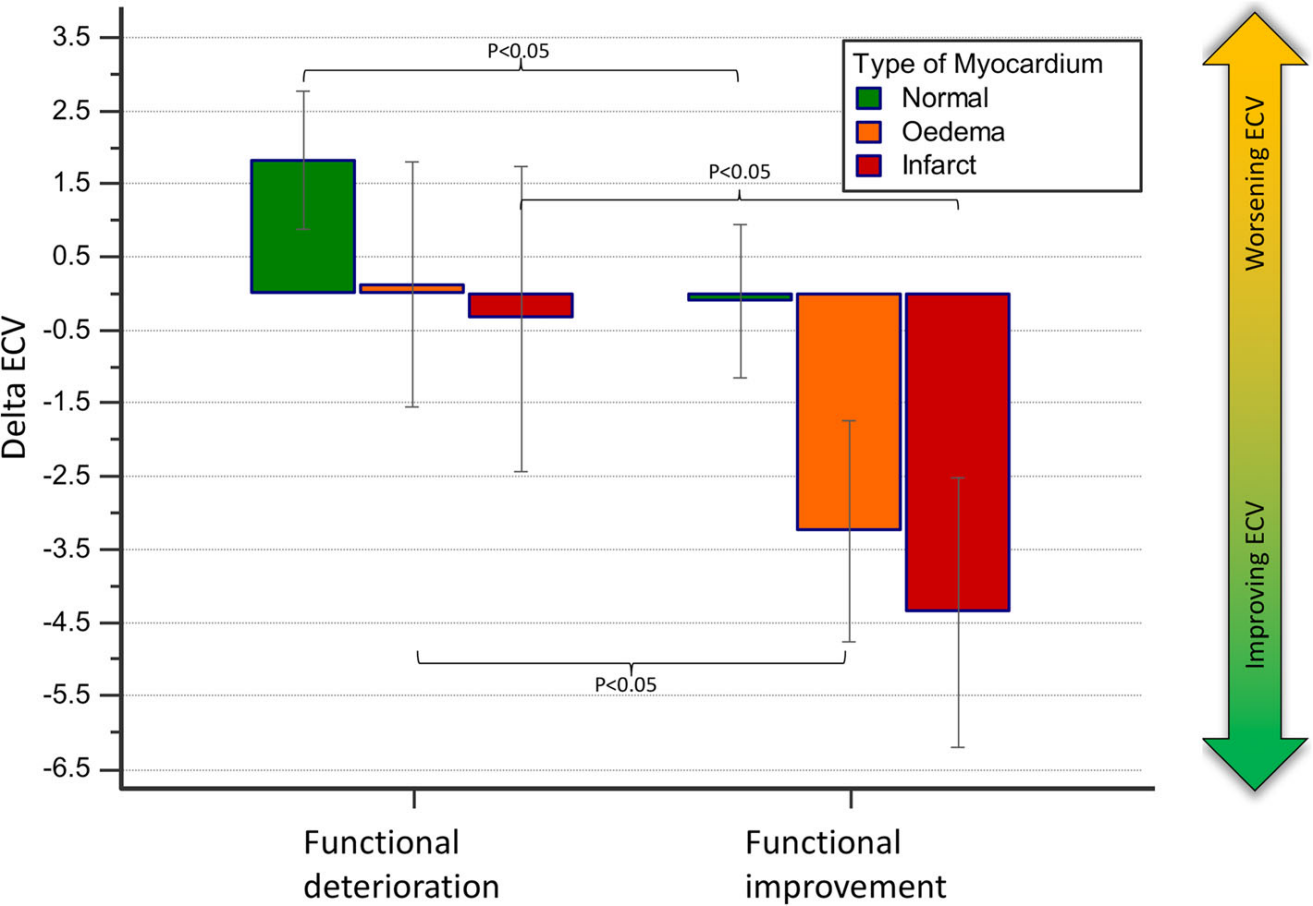
Frequency distribution histograms in normal, oedema and infarct segments with/without functional recovery from baseline to follow-up CMR

**Fig. 5:**
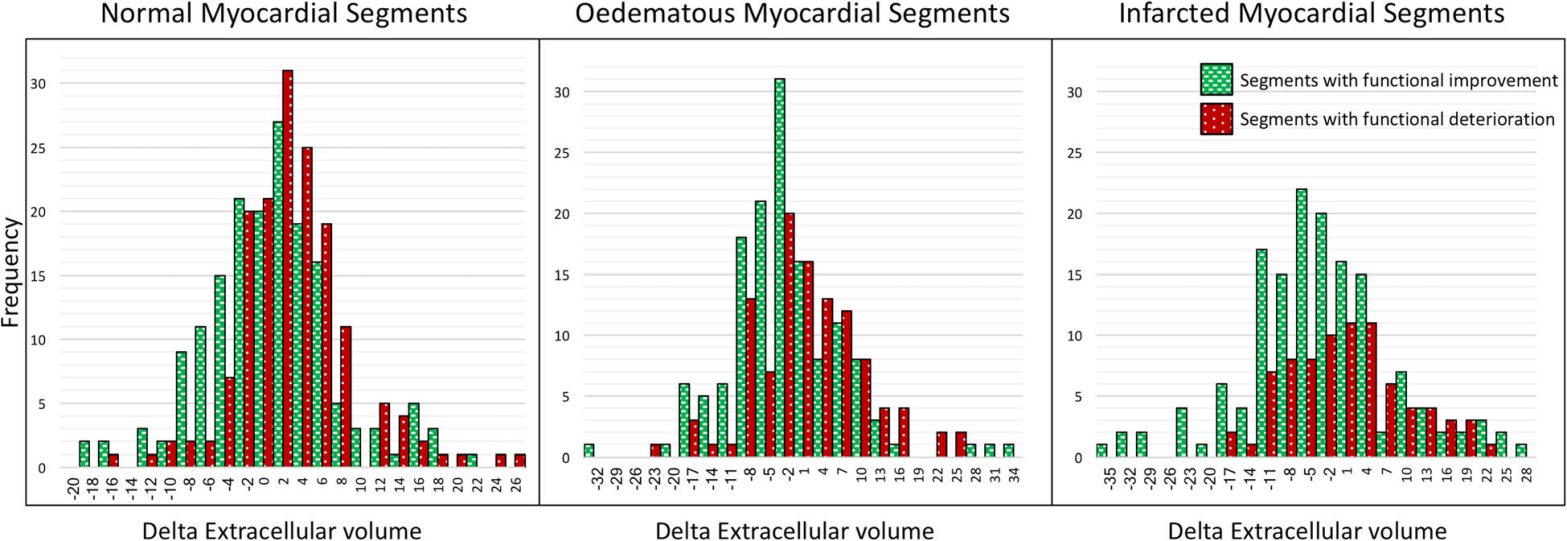
Box-and-whisker plot of temporal change in ECV of the normal myocardial segments and number of greater than 50 % transmural segments per patient

Acute segmental ECV demonstrated significant correlation to both acute segmental WT (*P* < 0.0001) and to follow-up segmental WT (*P* < 0.0001) (Table 3). There was a significant increase in ΔECV in normal segments which demonstrated deterioration in wall thickening (Table 3, Fig. 4). For the oedema and infarct segments, ΔECV was significantly different (less or no improvement) in segments with a decrease in wall thickening versus segments that demonstrated improvement in wall thickening (*P* < 0.05, Table 3, Fig. 5). All segments with functional improvement showed a reduction in follow-up ECV. The percentage of “normal” myocardial segments that demonstrated functional improvement and greater than normal acute baseline ECV (>28%) was not significantly different to “normal” segments with no functional recovery (54% versus 53%, *P* = 0.33).

**Table 3:** Segmental function and extracellular volume (ECV) results

Left Ventricular Segmental Function (800 segments)								
	Acute WT				Follow-up WT			
	Spearman rank correlation coefficient			*P*-value	Spearman rank correlation coefficient			*P*-value
Acute ECV	−0.46			<0.0001	−0.32			<0.0001
Follow-up ECV	−0.34			<0.0001	−0.35			<0.0001
		Function Deteriorated			Function Improved			
		Mean	SD	n (%)	Mean	SD	n (%)	
Delta ECV^a^ (%)	Normal	1.82	6.05	158 (19.8)	−0.10	6.88	167 (20.9)	*P* < 0.05
	Oedematous	0.12	8.72	107 (13.4)	−3.25	9.05	139 (17.4)	*P* < 0.05
	Infarct	−0.34	9.35	80 (10.0)	−4.36	11.42	149 (18.6)	*P* < 0.05

^a^Tests are adjusted for all pairwise comparisons within a row of each innermost sub-table using the Bonferroni correction

**Fig. 6:**
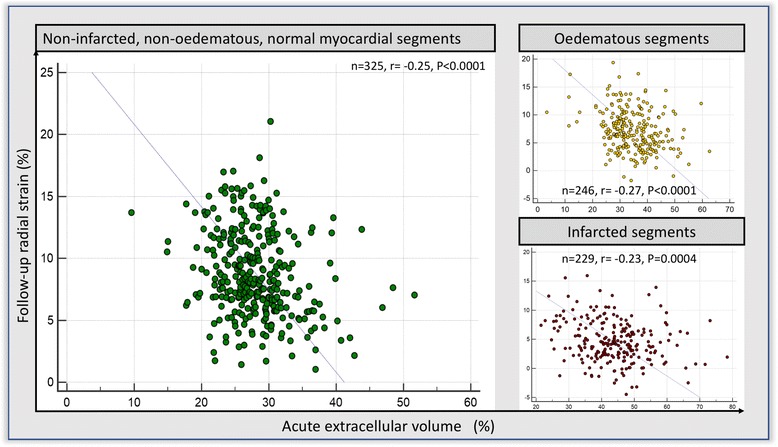
Scatter-plot of acute segmental ECV (x-axis) to follow-up peak systolic radial strain (y-axis)

Peak systolic radial strain results are detailed in the online **‘Supplementary material’**. Acute ECV of the normal, oedema and infarct segments demonstrated an inverse relation to final follow-up radial strain (Fig. 6).

By univariable analysis of all demographic and CMR parameters, acute oedema and infarct segment ECVs demonstrated association to number of segments (per-patient) that had improvement in function (Table 4). On multivariable linear regression analysis, infarct ECV was most strongly associated with number of segments with functional improvement (beta = 0.4, *P* = 0.037).

**Table 4:** Linear regression of baseline patient parameters to number of myocardial segments with improved function at follow-up study

Number of myocardial segments (per-patient) with improved function			
	Univariate	Multivariate	Beta
	*P*-Value	*P*-Value	
Age	0.11		
Sex	0.99		
Diabetes Mellitus	0.57		
Hypertension	0.69		
Smoker	0.53		
Time from onset of CP to reperfusion	0.93		
Killip Class I	0.76		
Killip Class II	0.85		
Killip Class III-IV	0.79		
Left main stem disease	0.14		
1-vessel disease	0.48		
2-vessel disease	0.67		
3-vessel disease	0.87		
TIMI flow pre-intervention	0.47		
TIMI flow post intervention	0.47		
LVEDV	0.74		
LVESV	0.76		
LV Mass	0.39		
LV ejection fraction	0.51		
Stroke volume	0.43		
Infarct size	0.57		
Area at risk (AAR)	0.18		
Acute non-infarcted normal myocardial ECV	0.89		
Acute AAR ECV	0.05	0.32	
Acute Infarct ECV	0.04	0.037	0.29

### Adverse LV remodelling

Acute myocardial ECV of normal segments was significantly higher in patients who demonstrated adverse LV remodelling (*P* = 0.04) (Table 5). Infarct and oedema acute ECV did not demonstrate a difference depending on the presence of adverse LV remodelling at follow-up.

**Table 5:** Association of acute myocardial ECV to adverse LV remodelling

	LV AVR -ve	LV AVR + ve	*P*-value
Number of patients (n)	40	10	
Acute non-oedematous, non-infarcted healthy myocardial ECV (%)	27.9 ± 3	30.4 ± 3.6	0.04
Acute infarct ECV (%)	43 ± 8	42 ± 6	0.61
Acute oedema ECV (%)	35.1 ± 6	33.3 ± 4	0.41

## Discussion

The present study demonstrates that 1) in reperfused STEMI, normal myocardial segments show a subtle expansion of the ECV between baseline and 3 month follow-up; 2) conversely, oedematous and infarcted segments show a significant reduction in ECV at follow up; 3) normal segments that demonstrate deterioration in segmental function at follow-up show a substantial increase in delta-ECV from baseline to follow up; 4) acute infarct ECV demonstrates the best association with the number of segments with functional recovery (Fig. 3) and 5) high acute normal myocardial segmental ECV is associated with adverse LV remodelling at follow-up.

Previous studies have already shown that ECV is raised in ‘remote’ myocardium in acute STEMI [26, 28]. Remote myocardium is defined as the AHA segment 180-degrees from the infarct territory with normal motion and no LGE [26, 28]. In the present study, we chose to assess all segments that did not have evidence of oedema or infarction and in order to avoid misinterpretation with the standard definition of ‘remote’ myocardium, defined these as ‘normal’ segments. Carberry et al. demonstrated that acute remote zone ECV post STEMI is associated with certain baseline patient characteristics (male gender, body mass index and history of diabetes) [28]. They also showed that the remote zone ECV was associated with the level of baseline N-terminal pro b-type natriuretic peptide (NT-proBNP). In our study the number of patients with angiographic triple vessel disease was marginally higher in patients with increased normal myocardial ECV when compared to patients with 0−/1−/2-vessel disease (Table 1, *P* = 0.05). We speculate that in triple vessel disease, coronary steal may reduce flow in non-culprit vessels due to better flow down the revascularised culprit vessel, which may cause adverse tissue level remodelling [29–31]. Other possible mechanisms which influence non-infarcted myocardium include increased loading conditions secondary to infarct characteristics. For example, Litwin et al.’s pre-clinical work demonstrated that the extra-cellular matrix of the non-infarcted normal myocardium undergoes expansion mainly due to increased wall stresses [6].

Associations of segmental ECV and segmental and global function have been studied previously [26, 32, 33]. Collins et al. demonstrated that the segmental extent of fibrosis is associated with segmental function in non-ischaemic cardiomyopathy [33]. Additionally, they demonstrated that this linear association was still relevant in patients with preserved global function and in patients with no late gadolinium enhancement. Our study has now shown that mean ECV is also associated with segmental function in patients with acute STEMI. Bulluck et al. demonstrated that mean segmental ECV in the remote myocardium is raised acutely post STEMI (18). Their mean segmental remote myocardial ECV values are comparable to the present study’s ‘normal’ myocardial segment ECV (27.9 ± 2.1%).

Remote (defined as normal myocardium in this study) myocardial dysfunction after STEMI is considered the main reason why some patients demonstrate function loss that is disproportionate to infarct size [30, 34]. Bogaert et al. demonstrated that remote myocardial dysfunction contributes significantly to the loss in global ventricular function. The present study confirms that normal myocardium with no obvious oedema or infarction can also be affected by functional loss measured by either LV wall thickening or radial strain. Additionally, this study further shows that normal myocardial segments after acute MI which demonstrate dysfunction also have significant extracellular matrix expansion. This could be explained by increased reactive fibrosis during the proliferative and maturation phase of remodelling, in the extracellular matrix of the normal myocardial segments, mainly due to higher wall shear stresses [35].

A recent study from our group demonstrated that acute ‘infarct zone’ ECV is predictive of regional and global LV functional recovery, and adds prognostic value over LGE [36]. Particularly for infarcts with higher transmural extent, acute infarct ECV was an additional predictor of functional recovery that can complement transmural infarct extent by LGE. In addition to these previously reported findings, we have now shown that acute infarct mean segmental ECV correlates with segmental functional recovery at follow-up and that a reduction in ECV in infarct segments is associated with improvement in regional function at follow-up.

**Fig. 7:**
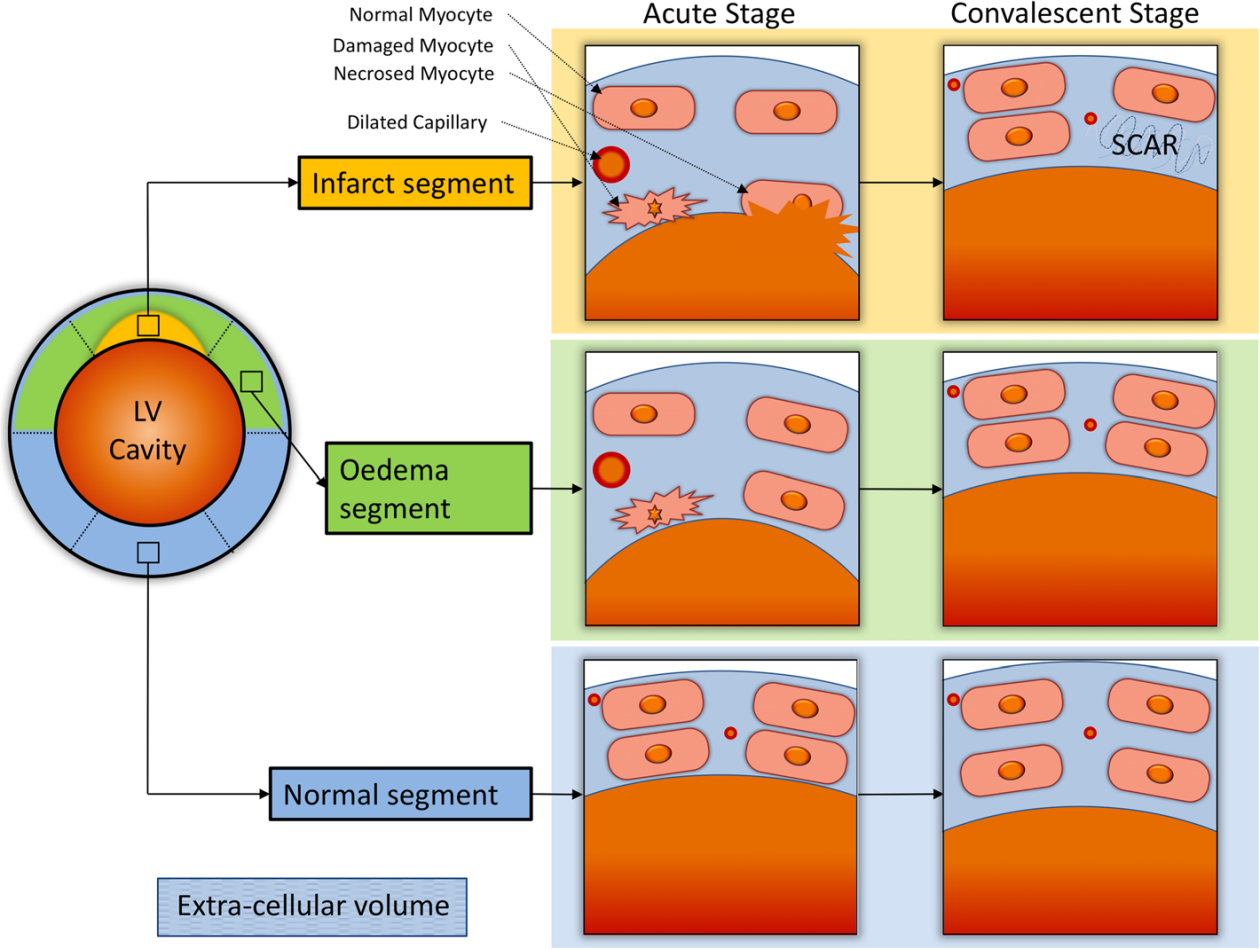
Illustration to demonstrate changes in tissue composition post-acute reperfused myocardial infarction. The infarcted myocardial segments demonstrate reduction in overall ECV on follow-up as the damaged myocytes recover function even in the presence of scar. This leads to some function improvement in these infarcted segments. The oedema segments demonstrate significant reduction in ECV as the water content of extracellular matrix falls. Oedema segments also demonstrate significant improvement in function. The normal segments undergo physiological adaptation with involves increase in extracellular volume. This is likely to be the result of overall left ventricular remodelling

This is the first in-vivo study that reports longitudinal changes in segmental ECV in non-infarcted normal, oedema and infarcted LV segments. Our data suggest that non-oedematous, non-infarcted normal LV myocardium undergoes remodelling with a subtle expansion of the extracellular matrix (Fig. 7). In particular, normal myocardial segments which demonstrate myocardial functional loss at follow-up have an increase in ECV.

### Limitations

Of 70 patients that underwent initial screening for recruitment only 50 patients underwent the entire protocol possibly introducing an element of selection bias. However, in a clinical study of acutely ill patients, exclusion or drop out of patients is common and our exclusion rate is within that reported in similar previous reports [9, 37]. There were other caveats to the present study: firstly, segments specifically classified as infarct also conceivably had oedema, which may have altered the results for respective segments. An important limitation of T1-mapping for clinical application is possible partial volume contamination from blood. Nevertheless, MOLLI sequences used in the present study, have been shown to be precise and reproducible [38]. The results of this study may be influenced by the tethering and local interaction of “adjacent” (to peri-infarct zone) normal myocardium. Changes in the adjacent normal myocardium may demonstrate a different pattern of temporal changes when compared to remote myocardium.

## Conclusion

This study suggests that following reperfused STEMI, the ‘normal’ LV myocardium undergoes remodelling with a subtle expansion of the extracellular matrix. In particular, normal segments which have functional loss demonstrate significant expansion of the extracellular space. Additionally, acute myocardial ECV of normal segments was significantly higher in patients who experienced adverse LV remodelling. Myocardial segments with oedema and infarction demonstrate significant reduction in ECV.

## Additional file

Additional file 1: Detailed methods. Comprehensive additional information on the basic CMR image analysis, T1-maps quality assurance checks, pulse sequence parameters and imaging protocol. (PDF 76 kb)

## Abbreviations

AHA: American Heart Association
ANOVA: a repeated-measures analysis of variance
CCC: concordance correlation coefficient
CMR: cardiovascular magnetic resonance
CV: coefficient of variability
ECV: extracellular volume
EDWT: end-diastolic wall thickness
EGE: early gadolinium enhancement
ESWT: end-systolic wall thickness
LGE: late gadolinum enhancement
LV: left ventricle/left ventricular
LVEDV: left ventricular end-distolic volume
MVO: microvascular obstruction
NT-proBNP: N-terminal pro b-type natriuretic peptide
PPCI: primary percutaneous coronary intervention
STEMI: ST-elevation myocardial infarction
T2 W: T2-weighted
TIMI: Thrombolysis In Myocardial Infarction
WT: wall thickening
