# Supplementary material for: Extra-cellular expansion in the normal, non-infarcted myocardium is associated with worsening of regional myocardial function after acute myocardial infarction
Source: J Cardiovasc Magn Reson. 2017 Sep 25;19:73. doi: 10.1186/s12968-017-0384-0 (PMC5613621; doi:10.1186/s12968-017-0384-0)
Supplement: Additional file 1: — Detailed methods. Comprehensive additional information on the basic CMR image analysis, T1-maps quality assurance checks, pulse sequence parameters and imaging protocol. (PDF 76 kb) [file 12968_2017_384_MOESM1_ESM.pdf]

Supplemental Material.

Image analysis

Cine, T2W-images, and LGE-images were evaluated offline using MASS research software (Version 2016EXP, Leiden University Medical Centre, Leiden, The Netherlands). Left ventricular volumes and EF were analysed from cine images using standard methods using endo/epi-contours at end-diastolic and end-systolic phases [1]. Infarct size (IS) was quantified using the previously published semi-automated full-width at half-maximum (FWHM) method on contiguous LGE short-axis slices [2,3]. Transmurality was assessed by measuring the depth of scar from the endocardium relative to the full thickness of the LV wall on LGE-imaging for all 800 segments on acute scans. Area at risk was similarly quantified using the previously validated FWHM method on T2-weighted short-axis contiguous slices [4]. MVO was defined visually as the hypo-intense core within the infarcted zone on EGE-imaging and planimetered manually. Additionally, radial strain was computed using endo-/epi- cardial contours throughout the complete cardiac cycle as per previously described methods [5].

T1-Maps quality checks

The presence of off-resonance artefacts and diaphragmatic movement was assessed by examination of the raw T1-weighted images by the radiographers. If significant off-resonance artefacts were detected live, T1-maps were repeated. T1-maps were generated on a Philips workstation.

We used parametric confidence maps of the goodness-of-fit generated (R2-maps) to assess how well T1-model fitting was achieved for each T1-map. Both the T1-maps and R2-maps were assessed by two independent assessors to check for partial volume effect and any other artefacts. In cases where there was partial volume effect, offline manual motion correction using MASS (Version 2016EXP, Leiden University Medical Centre, Leiden, The Netherlands) was applied to generate the maps. Care was taken to exclude epicardial structures and blood

pool from the endo/epi-cardial contours. Native T1 endo/epi cardial contours were transposed on post-contrast T1-maps manually and minor corrections applied if necessary for co-registration.

Table 1. Cardiac magnetic resonance study protocol sequence parameters for both field strength scanners.

MRI Systems	**1.5 Tesla** **(Philips Ingenia Omega HP)**	**3 Tesla** **(Philips Achieva TX)**
Cines (HLA, VLA, and full LV stack– contiguous slices)	• 30 phase bSSFP<br>• SENSE factor 2<br>• Typical TE/TR = 1.5/3.0 ms<br>• Flip angle = 60°<br>• Typical BW = 950 Hz/pix<br>• Typical in-plane resolution = 1.7 mm acq/1.0 mm recon<br>• Slice thickness = 8 mm	• 30 phase bSSFP<br>• No parallel imaging<br>• Typical TE/TR = 1.4/2.8 ms<br>• Flip angle = 45°<br>• Typical BW = 2500 Hz/pix<br>• Typical in-plane resolution = 1.7 mm acq/1.2 mm recon<br>• Slice thickness = 8 mm

MRI Systems	**1.5 Tesla (Philips Ingenia Omega HP)**	**3 Tesla (Philips Achieva TX)**
Native MOLLI T1-Map (3 of 5)	• IR bSSFP<br>• SENSE factor 2<br>• 5s(3s)3s scheme<br>• Typical TE/TR = 1.1/2.2 ms<br>• Flip angle = 35°<br>• Typical BW = 1100 Hz/pix<br>• Typical in-plane resolution = 2.0 mm acq/1.1 mm recon<br>• First TI after inversion from shortest possible value (typically 140 ms) to 350 ms<br>• Slice thickness = 10 mm.	• IR spoiled GE<br>• SENSE factor 2<br>• 3(3)3(3)5 scheme<br>• Typical TE/TR = 1.1/2.7 ms<br>• Flip angle = 35°<br>• Typical BW = 1100 Hz/pix<br>• Typical in-plane resolution = 1.7 mm acq/1.1 mm recon<br>• First TI after inversion from shortest possible value (typically 150 ms) to 350 ms<br>• Slice thickness = 10 mm.

MRI Systems	**1.5 Tesla (Philips Ingenia Omega HP)**	**3 Tesla (Philips Achieva TX)**
T2 Weighted (full LV stack – contiguous slices)	• SPIR TSE<br>• SENSE factor 1.8<br>• TE/TR = 75/2000 ms<br>• Flip angle = 90°<br>• Typical Bw = 500 Hz/pix<br>• Typical in-plane resolution =1.4 mm acq/0.8 mm recon<br>• Slice thickness = 8 mm	• SPIR TSE<br>• SENSE factor 2<br>• TE/TR = 90/1875 ms<br>• Flip angle = 90°<br>• Typical BW = 400 Hz/pix<br>• Typical in-plane resolution = 1.7 mm acq/1.4 mm recon<br>• Slice thickness = 10 mm

MRI Systems	**1.5 Tesla (Philips Ingenia Omega HP)**	**3 Tesla (Philips Achieva TX)**
EGE (3 of 5)	• IR spoiled GE<br>• SENSE factor 1.7<br>• Typical TE/TR = 2.8/5.7 ms<br>• Flip angle = 25°<br>• Typical BW = 300 Hz/pix<br>• Typical in-plane resolution = 1.5 mm acq/0.8 mm recon<br>• Look-Locker scout determined TI<br>• Slice thickness = 8 mm	• IR spoiled GE<br>SENSE factor 1.5<br>• Typical TE/TR = 2.0/3.8 ms<br>• Flip angle = 25°<br>• Typical BW = 700 Hz/pix<br>• Typical in-plane resolution = 1.5 mm acq/0.7 mm recon<br>• Look-Locker scout determined TI<br>• Slice thickness = 10 mm

MRI Systems	**1.5 Tesla (Philips Ingenia Omega HP)**	**3 Tesla (Philips Achieva TX)**
LGE (full LV stack – contiguous slices)	• PSIR spoiled GE<br>• SENSE factor 1.7<br>• Typical TE/TR = 3.0/6.1 ms<br>• Flip angle = 25°<br>• Typical BW = 250 Hz/pix<br>• Typical in-plane resolution = 1.6 mm acq/0.8 mm recon<br>• Look-Locker scout determined TI<br>• Slice thickness = 10 mm	• IR spoiled GE<br>• SENSE factor 1.5<br>• Typical TE/TR = 2.0/3.7 ms<br>• Flip angle = 25°<br>• Typical BW = 800 Hz/pix<br>• Typical in-plane resolution = 1.6 mm acq/0.7 mm recon<br>• Look-Locker scout determined TI<br>• Slice thickness = 10 mm

MRI Systems	**1.5 Tesla** **(Philips Ingenia Omega HP)**	**3 Tesla** **(Philips Achieva TX)**
Post-Contrast MOLLI T1-Map (3 of 5)	• IR bSSFP<br>• SENSE factor 2<br>• **4s(1s)3s(1s)2s scheme**<br>• Typical TE/TR = 1.1/2.2 ms<br>• Flip angle = 35°<br>• Typical BW = 1100 Hz/pix<br>• Typical in-plane resolution = 2.0 mm acq/1.1 mm recon<br>• First TI after inversion from shortest possible value (typically 140 ms) to 350 ms<br>• Slice thickness = 10 mm.	• IR spoiled GE<br>• SENSE factor 2<br>• **3(3)3(3)5 scheme**<br>• Typical TE/TR = 1.1/2.7 ms<br>• Flip angle = 35°<br>• Typical BW = 1100 Hz/pix<br>• Typical in-plane resolution =1.7 mm acq/1.1 mm recon<br>• First TI after inversion from shortest possible value (typically 150 ms) to 350 ms<br>• Slice thickness = 10 mm.

Abbreviations: bSSFP, MOLLI, MRI, HLA, VLA, LV, SPIR, SENSE, PSIR, BW, LGE, EGE, GE, IR

Table 2. Segmental radial strain at acute CMR and follow-study in the three categories of myocardial segments.

	**Radial Strain (%)**				
Type of segments	Acute		At follow-up		
	Median	25%-75%	Median	25%-75%	P-Value
Normal (n=325)	8.1	6-10.5	8.2	1-4.8	0.04
Oedema (n=246)	5.9	3.6-8	6.78	4.5-9.5	<0.0001
Infarct (n=229)	2.7	1.1-4.8	4.2	2.3-6.9	<0.0001

Reference:

1. Schulz-Menger J, Bluemke DA, Bremerich J, Flamm SDS, Fogel MA, Friedrich MGM, et al. Standardized image interpretation and post processing in cardiovascular magnetic resonance: Society for Cardiovascular Magnetic Resonance (SCMR) board of trustees task force on standardized post processing. J. Cardiovasc. Magn. Reson. BioMed Central; 2013;15:35.

2. Flett AS, Hasleton J, Cook C, Hausenloy D, Quarta G, Ariti C, et al. Evaluation of techniques for the quantification of myocardial scar of differing etiology using cardiac magnetic resonance. JACC. Cardiovasc. Imaging. 2011;4:150–6.

3. Amado LC, Gerber BL, Gupta SN, Rettmann DW, Szarf G, Schock R, et al. Accurate and objective infarct sizing by contrast-enhanced magnetic resonance imaging in a canine myocardial infarction model. J. Am. Coll. Cardiol. 2004;44:2383–9.

4. Aletras AH, Tilak GS, Natanzon A, Hsu L-Y, Gonzalez FM, Hoyt RF, et al. Retrospective determination of the area at risk for reperfused acute myocardial infarction with T2-weighted cardiac magnetic resonance imaging: histopathological and displacement encoding with stimulated echoes (DENSE) functional validations. Circulation. 2006;113:1865–70.

5. Russo C, Jin Z, Homma S, Rundek T, Elkind MS V, Sacco RL, et al. Relationship of multidirectional myocardial strain with radial thickening and ejection fraction and impact of left ventricular hypertrophy: a study in a community-based cohort. Echocardiography. NIH Public Access; 2013;30:794–802.
